# B602L-Fc fusion protein enhances the immunogenicity of the B602L protein of the African swine fever virus

**DOI:** 10.3389/fimmu.2023.1186299

**Published:** 2023-06-22

**Authors:** Yang Yang, Qiqi Xia, Lujia Zhou, Yan Zhang, Zhixin Guan, Junjie Zhang, Zongjie Li, Ke Liu, Beibei Li, Donghua Shao, Yafeng Qiu, Zhiyong Ma, Jianchao Wei

**Affiliations:** ^1^ Shanghai Veterinary Research Institute, Chinese Academy of Agricultural Sciences, Shanghai, China; ^2^ College of Animal Science and Technology & College of Veterinary Medicine of Zhejiang A&F University, Hangzhou, Zhejiang, China; ^3^ College of Veterinary Medicine, Nanjing Agricultural University, Nanjing, China

**Keywords:** African swine fever virus, B602L, IgG Fc, subunit vaccine, immunogenicity

## Abstract

African swine fever (ASF) is an acute, highly contagious, and deadly infectious disease caused by the African swine fever virus (ASFV) and has a huge impact on the pig industry. A lack of vaccines and effective therapeutic drugs has brought great challenges to the prevention and control of ASF. In this study, insect baculovirus expression system was used to express ASFV B602L protein (B602L) alone and the IgG FC-fused B602L protein (B602L-Fc), and evaluate the immune effect of B602L-Fc in mice model. To be specific, the ASFV B602L protein and B602L-Fc fusion protein were successfully expressed by the insect baculovirus expression system. Then, Functional analysis in vitro revealed that the B602L-Fc fusion protein bound and interacted with the FcRI receptor of antigen-presenting cells and significantly promoted the expression of proteins involved in antigen presentation and various cytokines at mRNA levels in porcine alveolar macrophages. Additionally, immunization using B602L-Fc fusion protein remarkably promoted the Th1-biased cellular immune response and humoral immune response in mice. In conclusion, The B602L-Fc fusion protein could up-regulate the expression of molecules involved in antigen presentation in APCs and enhance the humoral and cellular immune responses in mice. These results suggest that ASFV B602L-Fc recombinant fusion protein may be a promising candidate for subunit vaccine. This study provided useful data for the development of subunit vaccines for ASF.

## Introduction

African swine fever (ASF) is a highly infectious hemorrhagic and infectious viral disease of swine. There is currently no commercial vaccine to prevent ASF. Since the disease was first notified in 1921 in Kenya, ASF has spread to many African countries, affecting up to 35 African countries (1–3). In August 2018, China discovered its first case of ASF in Shenyang City, Liaoning Province, and it quickly spread to many parts of the country (4). At present, ASF continues to bring huge burden and economic losses to the pig industry in the epidemic area.

The cause of ASF is African Swine Fever Virus (ASFV), a large enveloped double-stranded DNA virus (5). The virus genome comprises single-molecule linear, covalent, closed double-stranded DNA (6). ASFV encodes more than 167 open reading frames and also encodes more than 170 proteins (7). At present, the structure and function of most ASFV proteins are still unknown (8). Currently, there are no commercialized ASF vaccines because of the complexity of the virus and the fact that the virus encodes multiple immune escape proteins. ASFV encodes a variety of proteins associated with immune evasion, and these viral proteins interfere with and down-regulate the expression of many host immunomodulatory proteins. Such as ASFV encoding A238L, MGF360,EP153R protein (5). The B602L protein of ASFV is encoded by the B602L gene; it is an important non-structural protein of ASFV and is the molecular chaperone of the p72 protein (9). B602L has a central variable region (CVR) that allows the subgenotyping of ASFV isolates based on this region (10, 11). B602L is highly antigenic (12); the protein can be taken as a candidate antigen for ASFV diagnosis, and domestic and wild boar immune sera can recognize the B602L protein (13, 14). Based on the above, the current research on B602L is also very important, and this protein may become a candidate antigen for ASFV detection and subunit vaccines.

So far, there are no commercially effective vaccines or drugs to prevent, control, and treat ASFV (15). ASFV vaccines currently under investigation include inactivated vaccines, live attenuated vaccines, and subunit vaccines. Inactivated vaccines cannot induce immune-protective responses such as cellular immune responses (16). Therefore, inactivated ASFV vaccine is basically an infeasible strategy. Safety of attenuated ASF vaccines remains challenging (17–19). Currently, the safest specific antigens vaccine are subunit vaccines, and ASFV subunit vaccines with specific antigens as the core have attracted wide attention. Proteins, such as p54, p30, p72, CD2v have been implicated in multiple steps of virus attachment and internalization (5). These proteins are considered immunogenic proteins, which can be used in subunit vaccine research; nonetheless, they do not produce complete protection (20). Recent research has shown that a variety of immunogenic ASFV proteins have been screened by prime booster vaccination. Pigs were immunized with three antigens p72, p54, and p12 and three modified vaccinia virus Ankara vector antigens p72, EP153R, and CD2v to induce ASFV-specific antibody and T-cell immune responses (21).

Therefore, the ASFV subunit vaccine has potential, but its immune protection effect need to be enhanced by some methods. On the other hand, besides neutralizing antibodies, cell-mediated immune response in the prevention of ASFV is also very important (3, 22). Oura et al. demonstrated that cell-mediated immune responses against the ASF virus are important (23). Vaccines that can induce humoral as well as cell-mediated immune responses are urgently needed to provide protection against ASFV.

The Fc receptors (FcγRs) of immunoglobulin G (IgG) belong to the immune receptor family and exist on the surface of various immune effector cells (23). FcRⅠ plays critical roles in the innate and adaptive immune responses by activating a variety of biological functions, such as processing and presentation of antigens, the release of inflammatory mediators, endocytosis of immune complexes, antibody protection against viral infection, microbial phagocytosis, and antibody-dependent cytotoxicity (24–26). Fc receptors are important mediators of cellular immunity and are also important receptors linking IgG-secreting lymphocytes and phagocytes (27). Fusion of antigens to Fc fragments is a new vaccine development strategy, which may cause Fc receptor-mediated antigen transport across the membrane barrier, thereby effectively enhancing the humoral and cellular immunities of the body (26). At present, studies on vaccine candidates have started using Fc fusion proteins, including pseudorabies virus (PRV) (gB-IgG2aFc) (28), human immunodeficiency virus (HIV) (Gag-Fc) (29), herpes simplex virus (HSV) (gD-Fc)(Ye, Zeng, Bai, Roopenian, & Zhu, 2011), classical swine fever virus (CSFV) (E2-Fc) (30). In recent studies, ASFV P30/P54 protein fused to porcine immunoglobulin Fc fragment induced the production of porcine P30/p54-specific antibodies and elicited stronger mucosal immunity in pigs, moreover the absorption and phagocytosis of recombinant *Saccharomyces cerevisiae* in IPEC-J2 cells or porcine alveolar macrophages (PAM) cells were also significantly enhanced (22). All these studies indicate that direct targeting of FcRⅠ receptors with fusion partners may provide research potential for Fc fusion proteins as vaccines.

In this study, ASFV B602L protein and a novel Fc refusion protein were expressed by the baculovirus expression system. We further examined whether ASFV B602L-Fc fusion protein can promote the immune response in mice. This study provides the basis and strategy for future research on ASFV subunit vaccine.

## Materials and methods

### Expression of African swine fever virus recombinant B602L and B602L-Fc fusion proteins in baculovirus

**Figure 1:**
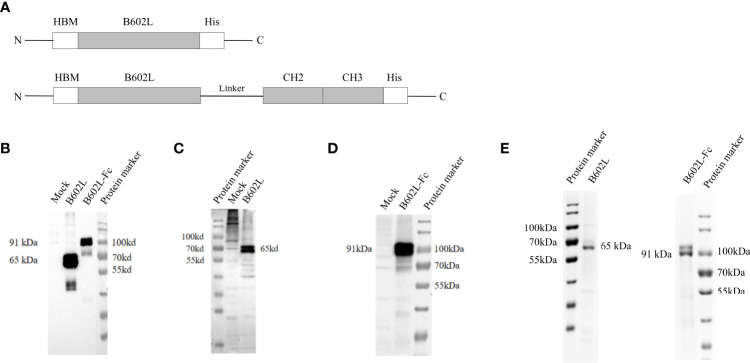
The construction and expression of recombinant ASFV B602L and B602L-Fc proteins. **(A)** Schematic diagram of recombinant protein construction. Schematic representation of HBM, B602L, Linker + CH2+CH3, and His-tag gene fusions constituting HBM-B602L-His and HBM-B602L-Linker + CH2+ CH3-His fusion proteins. **(B)** The expression of B602L and B602L-Fc protein was detected by an anti-His monoclonal antibody. Normal Sf9 cells, expression of B602L protein cells, expression of B602L-Fc protein cells, protein marker. **(C)** B602L protein expression was detected by porcine-positive serum. Protein marker, normal Sf9 cells, expression of B602L protein cells. **(D)** B602L-Fc fusion protein expression was detected by porcine-positive serum. Normal Sf9 cells, expression of B602L-Fc protein cells, protein marker. **(E)** Purification of B602L protein using nickel affinity column. protein marker, the purified protein B602L, the purified protein B602L-Fc, protein marker. The purity of purified B602L was 79.5%. B602L-Fc has a glycosylation site, so two bands of 91 kDa+ and 91 kDa presented after purification, and its purity was 85.9%.

ASFV-SY18 ((GenBank: MH766894.1)) B602L gene and the porcine IgG Fc gene synthesis and codon optimization were all outsourced by Tsingke Biotechnology (Shanghai, China), and cloned into pUC57 and verified by gene sequencing. Synthetic porcine IgG Fc fragment included hinge, extended CH2 and CH3 domains. The HBM signal peptide sequence was designed at the N-terminus of the B602L protein to promote protein expression (**Figure 1A**). The B602L and B602L-Fc tandem genes with 3×His tags were cloned into the baculovirus pFastBac expression vector, and the Bacmid plasmid was obtained by transposition. Bacmid plasmid was transfected into Sf9 cells to obtain baculovirus rBac-B602L and rBac-B602L-Fc which could express recombinant fusion protein. The detailed sequence of primers used in the construction process is shown in **Table 1**.

**Table 1:** Primers used for polymerase chain reaction assay.

Primer Name	Primer Sequence (5’-3’)
B602L-F 1	TTTCTTACATCTATGCGGATCGAATGGCAGAATTTAATAT
B602L-R 1	GACTGCAGGCTCTAGACTAGTGATGGTGATGGTGATGCAATTCTGCTTTTGTATA
B602L-F 2	TTTCTTACATCTATGCGGATCGAATGGCAGAATTTAATATTGATG
B602L-R 2	GAGCCACCTCCGCCTGAACCGCCTCCACCCAATTCTGCTTTTGTATATA
IgG Fc-F	GGTTCAGGCGGAGGTGGCTCTGGCGGTGGCGGATCGATCTGCCCCGCTTGCGAG
IgG Fc-R	GACTGCAGGCTCTAGACTAGTGATGGTGATGGTGATGCTTACCGGGGGTCTTACT

### Detection and purification of African swine fever virus B602L and B602L-Fc fusion proteins

The expression of ASFV B602L and B602L-Fc recombinant proteins was detected using indirect Immunofluorescence (IFA). Sf9 cells were infected with recombinant baculovirus rBac-B602L and rBac-B602L-Fc and placed at 28°C for 48 h in an incubator. The cells were fixed using 4% PFA at room temperature for 30 min, incubated with 5% bovine serum albumin for 30 min, and permeabilized with NP 40 solution for 15 min. Subsequently, the cell samples were incubated with ASFV-positive serum (1:1000, Qingdao, China) or anti-His monoclonal antibody (1:1000, Invitrogen, USA). Then, cells were treated with fluorescein isothiocyanate (FITC)-conjugated goat anti-pig antibody (1:400, ABclonal, China) or FITC-conjugated goat anti-mouse antibody (1:400, ABclonal, China). The nuclei were stained with DAPI (4’,6-diamidino-2-phenylindole). Lastly, the images results were taken under an inverted fluorescence microscope (Nikon).

ASFV B602L and ASFV B602L-Fc proteins were detected by western blotting. Sf9 cells were infected with B602L and B602L-Fc recombinant baculovirus and placed at 28°C for 72 h in an incubator, and then collected for western blot analysis. Protein samples were separated using 10% sodium dodecyl sulfate-polyacrylamide gel electrophoresis (SDS-PAGE) and transferred onto NC membranes. The NC membranes (nitrocellulose filter membrane) were incubated overnight at 4 °C with the ASFV positive serum (1:5000, Qingdao, China) and anti-His monoclonal antibody (1:5000, Invitrogen, USA) at 4 °C overnight respectively. The secondary antibody, the horseradish peroxidase (HRP)-labelled goat anti-pig antibody (1:5000, ABclonal, China) and HRP-labelled goat anti-mouse antibody (1:5000, ABclonal, China), were incubated at room temperature for 1 h and the signals were visualized using the ECL (Thermo, China) chemiluminescence system.

To purify B602L and B602L-Fc proteins, a large number of Sf9 cells were infected with recombinant baculovirus. After infection for 72 h, the cells were collected by centrifugation. ASFV B602L and B602L-Fc proteins were purified by BioLogic DuoFlow Chromatography System (Bio-Rid, USA) through a nickel affinity chromatography column. Lastly, the purified ASFV B602L and B602L-Fc proteins were identified by resolving them using 10% SDS-PAGE.

### Targeting of Fc fusion proteins

Preliminary verification of the Fc fusion protein function by western blot analysis and Immunofluorescence assay. For western blot analysis, we took the purified B602L and B602L-Fc proteins to prepare for gel SDS electrophoresis, transferred them onto NC membranes, blocked overnight at 4°C. The membranes were incubated with HRP-labelled goat anti-pig antibody (1:5000, ABclonal, China) and then with HRP-labelled goat anti-mouse antibody (1:5000, ABclonal, China) at room temperature for 1 h, and ETL (Thermo, China) was added for color development.

For the immunofluorescence assay, the Sf9 cells were infected with the recombinant baculovirus. The cells were fixed, blocked, punched and then directly incubated at room temperature for 1 h with FITC-conjugated goat anti-pig antibody (1:400, ABclonal, China) or FITC-conjugated goat anti-mouse antibody (1:400, ABclonal, China). The images were photographed under inverted fluorescence microscope (Nikon).

### B602L-Fc fusion protein binds to antigen-presenting cell FcRI receptor

The binding ability of B602L-Fc fusion protein to Fc receptor (FcRI) on APCs was determined. Therefore, the porcine alveolar macrophages (PAMs) were cultured in RPMI-1640 containing 10% fetal bovine serum in 24-well plates. When about 60-80% of the cells were adherent, B602L or B602L-Fc fusion proteins were added into the wells. After incubating at 37 °C for 12 h, the cells were fixed, blocked, and punched. The cells were incubated with anti-pig CD64 (FcRI) polyclonal antibody (1:1000, Absin, China) and anti-His monoclonal antibody (1:1000, Invitrogen, USA) for 60 min, sequentially. After that, the PAMs were incubated with FITC-conjugated goat anti-rabbit antibody (1:400, ABclonal, China) and FITC-conjugated goat anti-mouse antibody (1:400, ABclonal, China) for 60 min, respectively. The image results were taken under a laser scanning confocal microscope (LSM 510, Zeiss, USA).

### B602L-Fc fusion proteins related to the cells of porcine alveolar macrophage molecules

Porcine PAMs were prepared in a 24-well plate in a 37 °C cell incubator with 5% CO2. After the cells were adherent, purified B602L protein and B602L-Fc fusion protein were added, respectively and the cells were placed for about 12 h in the cell incubator; the wells supplemented with phosphate buffer saline (PBS) were set as a negative control. The medium supernatant was discarded, and cell samples were collected. The total RNA was extracted from the porcine PAMs cell samples and then analyzed by quantitative real-time polymerase chain reaction (q-PCR). **Table 2** shows primers used for quantitative RT-PCR. The 2^-ΔΔCT^ method was used to analyze the differences between the B602L group and the B602L-Fc group using GAPDH as the reference gene.

**Table 2:** Primers used for relative quantitative real-time polymerase chain reaction (RT-PCR).

Primer Name	Primer Sequence (5’-3’)
SLA-DM F	GGGACTCTCCGAGGTCTA
SLA-DM R	GTGAGCCCAGTCCGCAAA
CD80-F	GGTGTGGCCCAAGTATGA
CD80-R	CCTCTCTCCCGCTTCTGA
CD86-F	CTGGTGCTGCCTCCTTGA
CD86-R	ACCAGGTTATCCTGGTCC
CD40-F	TGCAGTGAAGGCCATCAC
CD40-R	GGGCAGGGTTCACAGAT
IL-1β-F	CCTTGAAACGTGCAATGATGACT
IL-1β-R	GTGGAGAGCCTTCAGCATGT
IL-6-F	CCGGTCTTGTGGAGTTTCAG
IL-6-R	CAGGGTCTGGATCAGTGCTT
IL-8-F	CCAGCATTCACAAGTCTTCTTGC
IL-8-R	ATGTCCTCAAGGTAGGATGGGG
IL-10-F	GCATCCACTTCCCAACCA
IL-10-R	TCGGCATTACGTCTTCCAG
IL-12(p40)-F	GTTTCAGACCCGACGAACTC
IL-12(p40)-R	GAGGACCACCATTTCTCCAG
IFN-α-F	ACTCCATCCTGGCTGTGAGG
IFN-α-R	CTTGCAGGTTTCTGGAGGA
IFN-β-F	TGCAACCACCACAATTCC
IFN-β-R	TCAAGTTCCACAAGGATAG
TNF-α-F	AGCCGCATCGCCGTCTCCTAC
TNF-α-R	CCTGCCCAGATTCAGCAAAGTCC
GAPDH-F	TCTGGCAAAGTGGACATT
GAPDH-R	GGTGGAATCATACTGGAACA

Data statistics and analysis: GraphPad Prism 8 was used to make a statistical chart, and the two-tailed T-test was used to analyze the significance of the difference between the B602L and B602L-Fc experimental groups. *p <*0.05 and *p <*0.01 were considered significant, * and ** respectively represent *p <*0.05, *p* < 0.01.

### Immunization of mice

**Figure 2:**
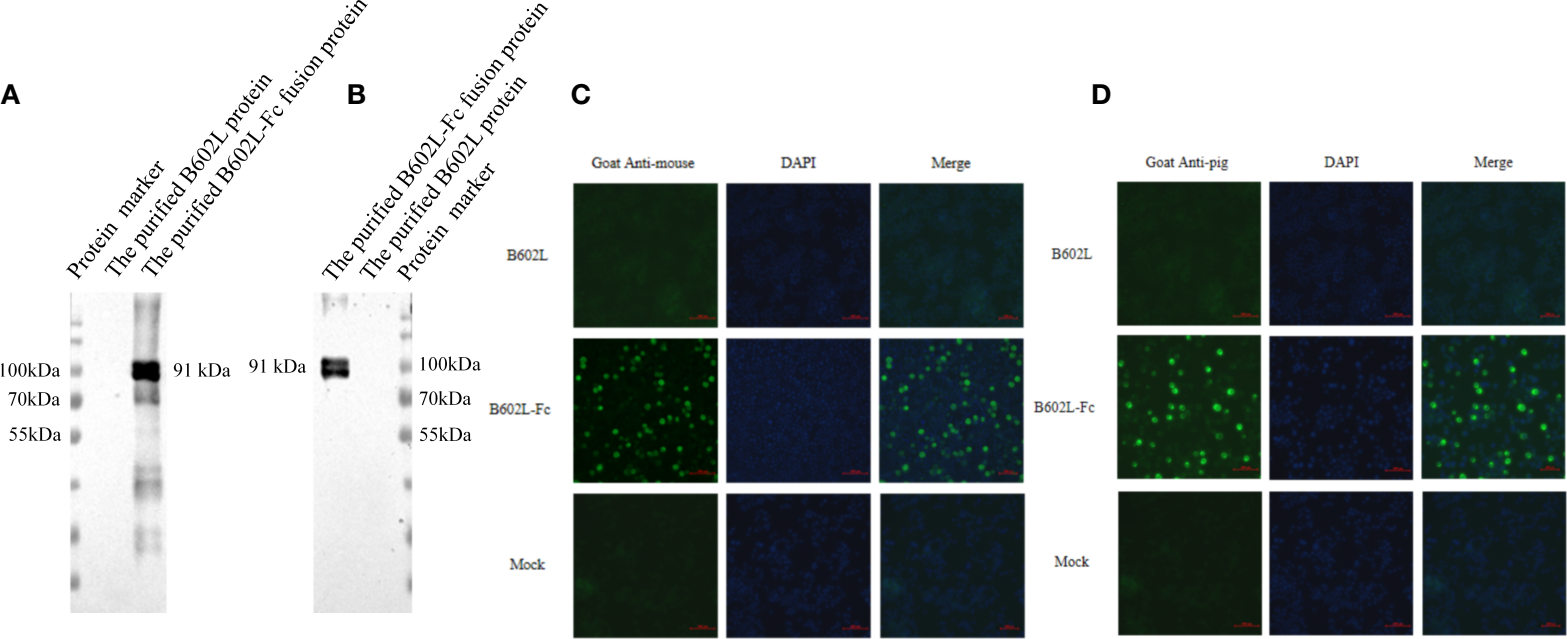
The B602L-Fc fusion protein can be identified by a secondary antibody by **(A)** horseradish peroxidase (HRP)-labeled goat anti-mouse secondary antibody; **(B)** HRP-labeled goat anti-pig secondary antibody; **(C)** fluorescein isothiocyanate (FITC)-labeled goat anti-mouse secondary antibody; and **(D)** FITC-labeled goat anti-pig secondary antibody. Detection of B602L-Fc using FITC 488 goat anti-pig antibody shown as green fluorescence. The nuclei (blue) were labeled with DAPI. Scale bars,100 µm.

The female Balb/c mice aged 4 weeks were bought from the JSJ (Shanghai, China) and randomly categorized into three groups (n = 10/group). Afterwards, the mice were subcutaneously immunized with the same dose (200 pM) of B602L (Group A), B602L-Fc (Group B), and (200 μL) PBS (Group C). For the first immunization, the purified proteins were emulsified in a 1:1 (w/w) ratio using Freund’s complete adjuvant (Beyotime, China). Each mouse received subcutaneous multipoint injection of immune protin. After three weeks of the first vaccination, each mouse was injected with the same dose (200 pM) of protein at subcutaneous multipoint injection. The proteins were emulsified with Freund’s incomplete adjuvant (Beyotime, China) in a ratio of 1:1 (w/w). Body-weight changes of mice immunized with B602L and B602L-Fc protein were monitored for 49 days. The blood samples were collected from orbital veins at 0, 7, 14, 21, 28, 35, 42, and 49 days after the primary immunization, and serum was then isolated for the detection of specific antibodies. On day 49 of immunization, three mice in each group were randomly sacrificed to isolate spleen lymphocytes aseptically to detect lymphocyte proliferation. The immunization procedure and regimen of mice is shown in **Table 3** and **Figure 2**. After the end of the experiment, all the mice in the experiment were euthanized, which  was in accordance with the ethics for the treatment of experimental animals.

**Table 3:** Immunization procedure of mice.

Group	Immunization dose	Number of mice	Immunization Times	Immune route
B602L	200 pM/200 µL	10	2	Subcutaneous multipoint injection
B602L-Fc	200 pM/200 µL	10	2	Subcutaneous multipoint injection
PBS	200 µL	10	2	Subcutaneous multipoint injection

### Determination of specific antibodies in mouse serum

ASFV B602L specific antibody was detected by enzyme linked-immunosorbent assay (ELISA) in each group at 0, 7, 14, 21, 28, 35, 42, and 49 days after the first immunization (31). Indirect ELISA was used to detect mice serum ASFV B602L specific antibodies endpoint titers at 14, 28, and 42 days. The indirect ELISA method and the pB602L protein previously constructed in our laboratory was used to detect serum antibodies (31). Briefly, the pB602L protein was mixed with coating buffer (pH 9.5) in 96-well plates and coated overnight at the protein concentration of 0.04 µg/mL per well. The plates were blocked with 2% bovine serum albumin at 37 °C for 2 h. The serially diluted serum was added and incubated for 1 h at 37°C. Next, HRP-conjugated goat anti-mouse IgG (1:5000) (ABclonal, USA) was added and incubated at 37 °C for 1 h. Finally, the absorbance was measured at OD450 mm on a microplate reader.

According to the above method, 14 -, 28 -, and 42-day mouse serum was added with a two-fold serial dilution starting at 1: 100 up to 1:51200 was used. Finally, the absorbance was measured at OD450mm on a microplate reader.

### Lymphocyte proliferation analysis

After 49 days of first immunization, three mice in each group were randomly selected, and the spleens of mice were collected aseptically. Then, the spleen lymphocytes of immunized mice were isolated according to the mouse spleen lymphocyte isolation kit (TBD, China), and lymphocytes (1×10^6^ cells/mL) were cultured with 100 μL RPMI-1640 containing 10% fetal bovine serum in 96-well plates. To stimulate these lymphocytes, RPMI-1640, concanavalin A (5 μg/mL), and purified pB602L protein (10 μg/mL) were added to each well. After culturing at 37 °C for 72 h, 20 μL CCK-8 solution was added to each well. The incubation was then continued at 37°C for 3 h to calculate the stimulation index for each group. The stimulus index (SI) was calculated as follows: SI = (OD values of immunized groups – OD values of blank control)/(OD values of the negative control group – OD values of blank control).

### Cytokine assays

The spleen lymphocytes of immunized mice were isolated according to the mouse spleen lymphocyte isolation kit. Isolated mouse lymphocytes were specifically stimulated *in vitro* using purified B602L protein, and stimulated cell supernatants were collected after incubating at 37 °C for 48 h. Then, the corresponding ELISA kits (CUSABIO, USA) were used for the detection of interferon (IFN-γ) and interleukin (IL-4) levels. Finally, the concentrations of different cytokines were measured according to the standard curve.

### Statistical analysis

All data were analyzed using GraphPad Prism 8 software (GraphPad Software, La Jolla, CA, USA). The two-tailed Student’s t-test was used to analyze all data. *p* < 0.05 was considered statistically significant.

## Results

### Expression of African swine fever virus recombinant B602L and B602L-Fc fusion proteins

The expression of ASFV B602L and B602L-Fc recombinant proteins was detected by immunofluorescence assay (IFA). Green fluorescence signal was detected in Sf9 cells infected with B602L and B602L-Fc recombinant baculovirus but not in normal cells when ASFV-positive serum or anti-His monoclonal antibody were used as primary antibodies (**Supplementary Figure 1**). Then, the expression of ASFV-B602L and ASFV-B602L-Fc recombinant proteins was identified in recombinant baculovirus by western blot analysis. The results showed that the target bands at 65 and 91 kDa were detected with ASFV anti-His monoclonal antibody (**Figure 1B**) or positive serum in Sf9 cells, respectively (**Figures 1C, D**). These results confirmed the expression of ASFV-B602L and ASFV-B602L-Fc proteins.

Then, the ASFV B602L and B602L-Fc proteins from Sf9 cells were purified using nickel affinity column and the purified recombinant proteins were verified using 10% SDS-PAGE (**Figure 1E**).

According to the results of western blot analysis and SDS-PAGE, two bands of similar size were found for B602L and B602L-Fc proteins. The B602L and B602L-Fc proteins were found to possess putative glycosylation sites; thus, the two bands detected by western blotting and SDS-PAGE may be due to glycosylation and other post-expression modifications during baculovirus expression.

### Preliminary functional verification of the Fc fusion protein

**Figure 3:**
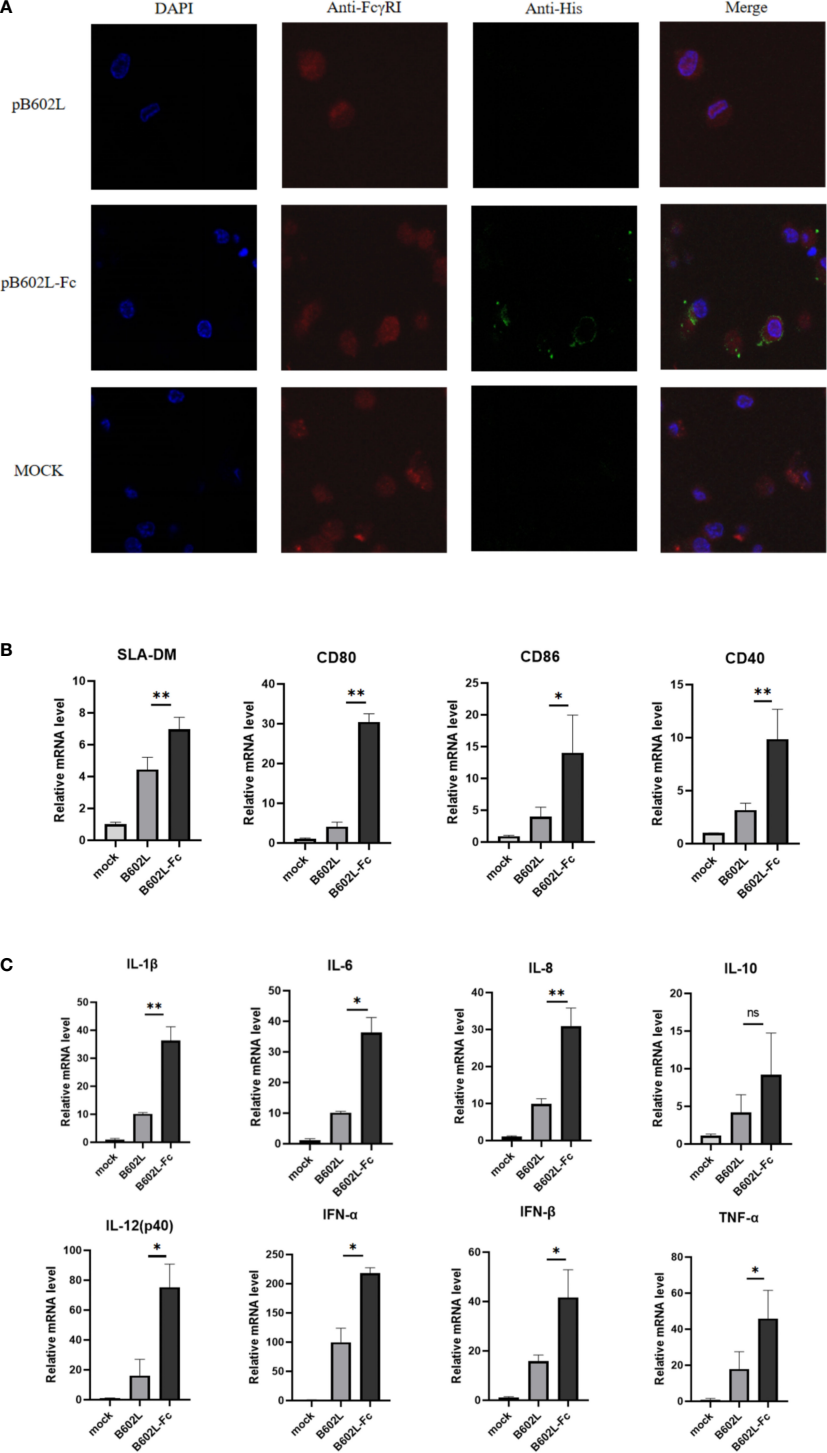
The B602L-Fc fusion protein promoted antigen presentation. **(A)** Co-localization of FcγRI on macrophages and ASFV B602L-Fc fusion protein. Confocal visualization of the subcellular localization of pAb CD64 (FcRI) and mAb His in macrophages. Immunofluorescent microscopic photomicrographs displaying localization of FcRI on macrophages, and the pAb FcRI (CD64)-immunoreactive red fluorescence was detected by FITC-conjugated 594 goat anti-rabbit antibody. The localization of B602L in FcRI was analyzed through the mAb His-immunoreactive green fluorescence using FITC-conjugated 594 goat anti-mouse antibody. The nuclei (blue) were labeled with 4′,6-diamidino-2-phenylindole (DAPI). Each image was merged to analyze the subcellular localization of CD64 (FcRI) and B602L in macrophages. Scale bars, 5 µm. **(B)** Effect of B602L-Fc fusion on mRNA expression of antigen-presenting molecules (SLA-DM, CD80, CD86, and CD40) in porcine PAMs. Purified B602L and B602L-Fc protein were used to stimulate PAMs, respectively, and the negative control group was added with the same amount of PBS to stimulate PAMs. **(C)** Effect of B602L-Fc fusion on mRNA expression of cytokines in porcine alveolar macrophages. Interleukin (IL)-1β, IL-8, IL-6, IL-12 (P40), IL-10, interferon (IFN)-α, IFN-β and tumor necrosis factor (TNF)-α. Purified B602L and B602L-Fc protein were used to stimulate PAMs, respectively, and the negative control group was added with the same amount of PBS to stimulate PAMs. ns *p*>0.05, *p<0.05 and **p<0.01.

The function of the Fc fusion protein was preliminarily verified by western blot analysis. B602L-Fc fusion protein could be recognized by HRP-labeled goat anti-mouse secondary antibody (**Figure 3A**) and goat anti-pig secondary antibody (**Figure 3B**). B602L-Fc protein showed a target band at 91 kDa, while B602L protein did not show the expected target band. These results indicated that the IgG Fc segment of B602L-Fc fusion protein expressed and purified in this study could bind directly to murine and porcine secondary antibodies.

**Figure 4:**
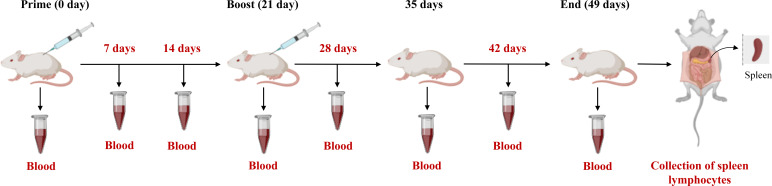
The immunization procedure and regimen of mice.

IFA assay was used to preliminarily verify the function of the Fc fusion protein and the results are shown in **Figure 4**. B602L-Fc fusion protein could be recognized by FITC-labeled goat anti-mouse secondary antibody (**Figure 3C**) and goat anti-pig secondary antibody (**Figure 3D**); the cells showed green fluorescence, while B602L protein did not show fluorescence. The result was consistent with those of western blotting assay. Western blotting and IFA assays indicated that the Fc segment of B602L-Fc fusion protein expressed and purified in this study has the function of directly binding to murine and porcine secondary antibodies.

### B602L-Fc fusion protein bindings to FcRI receptors on antigen presenting cells

Laser confocal analysis was used to determine the binding of the Fc fusion protein (B602L-Fc) to the FcRI receptor on APCs. B602L-Fc fusion recombinant protein could bind to FcRI on porcine macrophages (**Figure 4A**). The confocal results of immunofluorescence analysis showed that green fluorescence was not detected in the B602L protein incubation group, while green fluorescence was detected in the B602L-Fc fusion protein incubation group, indicating that B602L-Fc fusion protein could bind to FcRI receptors of PAM cells, and the fusion protein had the ability to target APCs.

### The effect of B602L-Fc fusion protein on the production of antigen-presenting molecules and cytokines in porcine alveolar macrophages

To determine whether endocytosis induced by the binding of B602L-Fc fusion protein to FcRI receptor would promote the production of molecules and cytokines involved in antigen presentation after the occurrence of endocytosis, relative fluorescence quantitative PCR was performed for molecules related to antigen presentation and cytokines. The results showed that B602L-Fc protein could promote the mRNAs expression levels of antigen-presenting molecules, SLA-DM, CD80, CD86, and CD40, while the mRNA levels of the B602L protein group were basically unchanged. Compared with the B602L group, the mRNA levels of SLA-DM, CD80, and CD40 (*p <*0.01), and that porcine alveolar macrophages of CD86 were significantly different (*p <*0.05) (**Figure 4B**). Cytokine detection showed that B602L-Fc protein could stimulate the increase in mRNA expression of cytokines IL-1β, IL-8, IL-6, IL-12 (P40), IL-10, IFN-α, IFN-β, and TNF-α. Compared with the B602L protein group, the mRNA levels of IL-1β and IL-8 were significantly different (*p <*0.01), and that of IL-6, IL-12 (P40), IFN-α, IFN-β, and TNF-α were also significantly different (*p <*0.05) (**Figure 4C**). These results indicate that B602L-Fc fusion protein can stimulate the production of molecules related to antigen presentation (SLA-DM, CD80, etc.) and cytokines (IL-1β, IL-8, IL-6, etc.).

### The B602L-Fc fusion protein to enhance the humoral immune response

**Figure 5:**
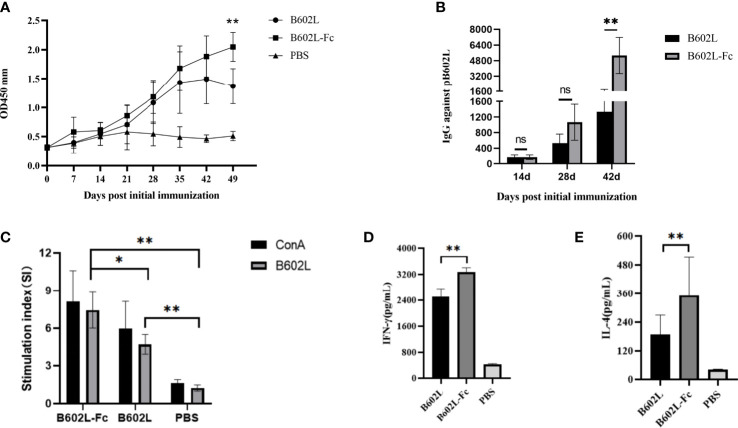
B602L protein and B602L-Fc fusion protein immunized mice with humoral immunity and cellular immunity. **(A)** After primary immunization, B602L-specific IgG levels were determined by indirect ELISA at 0, 7, 14, 21, 28, 35, 42 and 49 days. **(B)** Detection of B602L-specific IgG titers in serum samples by indirect ELISA after 14, 28, and 42 days of primary immunization. **(C)** Lymphocyte proliferation was tested by the CCK8 lymphocyte proliferation assay at 49 d after primary immunization. **(D, E)** At day 49 after the first immunization, a cytokine ELISA Kit was used to detect the levels of cytokines IFN-γ and IL-4 in the supernatant of stimulated lymphocytes *in vitro* ns *p*>0.05, *p<0.05 and **p<0.01. ELISA, enzyme-linked immunosorbent assay; IFN, interferon; IL-4, interleukin 4.

To evaluate the effect of B602L-Fc fusion protein on humoral immunity response, the levels of B602-Fc specific mouse serum antibodies at different times were measured using an in-house indirect ELISA, and the result was shown in **Figure 5**. The body-weight change of the immunized mice were monitored for 49 days. The body weight of B602L and B602L-Fc protein immunized mice showed no difference from that of PBS immune mice, and their body weight increased normally (**Supplementary Figure 2**), indicating that these two proteins have high immune safety. Specific antibodies were produced in both the B602L protein immunized group and the B602L-Fc protein immunized group after 0-21 days of the initial immunization. Further, after the booster immunization, the antibody levels of the two groups were significantly increased. The specific antibody production rate of the B602L-Fc group was faster than that of the B602L group (**Figure 5A**). After 21 days of primary immunization (after enhanced immunization), the level of specific antibody in the B602L-Fc group was higher than that in B602L group, with a faster production rate (**Figure 5A**). At 42 days after primary immunization, the antibody level of the B602L-Fc group tended to be stable, while the antibody level of B602L group may start to decrease, indicating that the antibody level in the B602L-Fc group lasted longer (**Figure 5A**). During this process, no specific antibody was produced in the PBS group. The results of specific antibody titer levels in serum of mice after 14, 28, and 42 days of the first immunization are shown in **Figure 5B**. At 14 days after the first immunization, the antibody levels of the B602L and B602L-Fc groups were lower, and the antibody titers of the two groups were similar. At 28 days after the first immunization, the serum-specific antibody titer of the B602L-Fc group was higher than that of the B602L group, and there was no difference in antibody titers between 14d and 28d. However, at 42 days of immunization, the serum-specific antibody titer of the B602L-Fc group was significantly higher than that of the B602L group (**Figure 5B**). To sum up, the above results suggest that the B602L-Fc fusion protein enhanced the immunogenicity of ASFV B602L protein and effectively improved the antibody titers, thus effectively inducing and enhancing the humoral immunity level.

### The B602L-Fc fusion protein to enhance cellular immune responses

To further investigate the effect of B602L-Fc fusion protein on cellular immune response, lymphocyte proliferation was first detected. The stimulus index (SI) of the two protein immunization groups was significantly different from that of the PBS group. Moreover, the SI of the B602L-Fc group was significantly higher than that of the B602L group, with a statistically significant difference (*p <*0.05) (**Figure 5C**). Next, detection of intracellular cytokines IL-4 (induced by the Th2 cellular response) and IFN-γ (induced by the Th1 cellular response) by ELISA suggested increased secretion of IL-4 and IFN-γ in the immune protein group. Additionally, the secretion of IL-4 and IFN-γ in the B602L-Fc fusion protein group was significantly higher than in the B602L protein group (**Figures 5D, E**). Hence, these findings revealed that Fc fusion protein could significantly enhance cellular immune responses.

## Discussion

The viral genome of ASFV is extremely complex, and also encodes a variety of proteins associated with immune evasion. Therefore, development of a safe and effective ASF vaccines remains a challenging task (32). At present, subunit vaccine has high safety, but their protective efficacy is not very ideal. Thus, it is necessary to develop more immunological protective antigens or combinations or accelerate the recognition and presentation of antigens and generate a humoral and cellular immune response quickly to produce a stronger immune protective effect (5, 6). DNA vaccines show better immunogenicity because the antigen is expressed intracellularly and presented via MHC-I, which is essential for CD8^+^ T cell activation (33). IgG Fc fusion proteins have been shown to accelerate antigen presentation and generate higher levels of immunity, displaying excellent potential for use in vaccine development (22, 28, 34).

In the present study, the recombinant fusion proteins of ASFV B602L and B602L-Fc were expressed and purified using an insect baculovirus expression system. ASFV B602L-Fc protein was found to combine with FcRI on PAMs and enhance the expression of antigen presentation-related molecules and cytokines of APC. Moreover, the ASFV-B602L-Fc protein enhanced humoral immunity levels and specific T cell immune response in mice.

The baculovirus expression vector systems have been used to produce commercially available vaccines (35, 36). In previous studies on ASFV subunit vaccines, the baculovirus expression system was used to express p54, p30, p72, and other proteins, and certain immune effects could be achieved (37). Consequently, in this research, the baculovirus expression system was selected to express the required fusion protein.

ASFV B602L, the major non-structural protein of ASFV, is also known as the chaperone protein of p72, essential for the formation of the icosahedral capsid of ASFV. ASFV B602L protein was recognized by the immune sera of domestic pigs and wild boar and showed good antigenicity and immunogenicity (13, 14). Lokhandwala et al. (2017) evaluated the immunogenicity of seven novel ASFV antigens from adenovirus vectors, including B602L. The recombinant adenovirus mixture elicited strong humoral and cellular immune responses in immunized pigs; moreover, the B602L-induced antibody production was higher than that in the control group (14). Goatley et al. showed that specific antibodies against B602L could be produced using a cocktail of adenovirus and poxvirus vectors with different ASFV antigen pools (including B602L); the mixed immunization could indeed produce a better immune protection effect (38). Therefore, in this study, the ASFV B602L protein and the B602L-Fc fusion protein were successfully expressed using the baculovirus expression system. B602L and B602L-Fc proteins could be recognized by anti-His and ASFV positive sera, indicating good antigenicity of the expressed proteins.

Recombinant proteins expressed by Fc fusion may promote antigen recognition and antigen phagocytosis, thereby accelerating antigen presentation and improving immune efficiency (39, 40). The Fc region displays powerful immune effector functions through its involvement in FcRI and serum complement proteins (28). Porcine IgG Fc may bind porcine FcRI receptors; FcRI is ubiquitous on the surface of APCs, including dendritic cells and macrophages, and IgG Fc binding to FcRI receptors could enhance immune response (39, 41). The Fc fusion protein is attracting increasing attention as an increasingly safe and useful therapeutic protein (42). The main target cells of ASFV are mononuclear macrophages. Interestingly, in our research, the B602L protective antigen genes were linked to the porcine IgG Fc gene (including hinge region, CH2, and CH3 domains) to construct a recombinant baculovirus expression system capable of expressing B602L-Fc. The findings suggest that the novel subunit vaccine model in which pig IgG Fc fragment and ASFV protein were connected in series to form Fc fusion protein was indeed established.

The optimal binding of Fc-fusion proteins to one or more of FcRI, which is expressed on the surface of APCs, enhances the uptake of antigens by APCs (34, 39). In this study, the western blotting and IFA detection results of incubation of only sheep anti-mouse and sheep anti-pig secondary antibodies showed that the purified B602L-Fc fusion protein could be directly recognized by the secondary antibodies, while the B602L protein was not recognized. These results indicated that the Fc segment of the B602L-Fc fusion protein could directly bind to the secondary antibody. The Fc fusion recombinant protein (B602L-Fc) was identified to bind to Fc receptors (FcRI) on PAMs. These findings suggest that the B602L-Fc protein may promote phagocytosis of APCs, which is then processed and presented or cross-presented to T cells, thereby enhancing the adaptive immune response.

APCs produce immune effector molecules and effector cells after antigen recognition, processing and presentation (43). Antigen processing, and presentation are not only completed by APCs but also involve many functional molecules; the more important ones are MHC molecules, chaperone molecules, and costimulatory molecules (44). Macrophages are important APCs, and are also important target cells of ASFV. PAMs must simultaneously express sufficient amounts of MHC molecules, SLA-DM molecules, CD80, CD86, and CD40 molecules to complete an effective antigen presentation process and immune response (45, 46). Therefore, we hypothesized that when B602L-Fc occurs through FcRI-induced endocytosis, a variety of antigen presentation signaling pathways are triggered by Fc receptor cross-linking and phagocytosis, resulting in the production of antigen-presenting molecules and cytokines (30). Previous studies have shown that Fc fragments of two Fc-fused ASFV proteins can promote the adhesion and phagocytosis of PAM cells to foreign particles, thereby accelerating antigen presentation and improving immune efficiency (22). Interestingly, in this study, we observed that B602L-Fc could stimulate the increase in antigen-presenting molecules (SLA-DM, CD80, CD86, and CD40) and cytokines (IL-1β, IL-8, IL-6, IL-12 (P40), IL-10, IFN-α, IFN-β, and TNF-α) in PAMs at the mRNA level. The current study results show that B602L-Fc can enter cells through Fc receptor-mediated endocytosis and activate a series of signaling pathways to initiate phagocytosis. The production of these molecules is beneficial to the macrophage activation and the phagocytosis of foreign antigens. In short, recombinant Fc fusion proteins may promote the binding and endocytosis of porcine -derived cellular antigens, thereby accelerating antigen presentation and improving immunological efficiency.

Antibody-mediated humoral immune response and cellular immune response mediated by CD8^+^T cells play key roles in protective immunity (5, 23). The specific immunity level induced by the vaccine was measured from the humoral immune response and cellular immune response. Therefore, in this study, the humoral immune response was evaluated mainly at the level of specific antibodies in serum, and the cellular immune response was evaluated at levels of lymphocyte proliferation and the cytokines (IFN-γ and IL-4). Mouse and pig IgG Fc fragments have similar homology, and the previous study showed that the pig Fc fragment selected in this study could directly bind the murine secondary antibody; therefore, so mice were selected for animal experiments in this study. Our results showed that both the B602L protein and B602L-Fc protein group could induce specific antibodies. The rate of antibody induction and expression by the B602L-Fc protein group was higher; the antibody titer of the B602L-Fc protein group was significantly higher than that of the B602L protein group at 42 days. Furthermore, one of the important methods to improve the stability of protein and obtain long-lasting recombinant protein is the fusion expression of antigen protein and Fc fragment of IgG antibody (47). FcRⅠ plays primary roles in the half-life of IgG, regulating the catabolism and its functions in the immune system, and especially the presentation of complexed antigens (42). We also observed a longer duration of antibody generation induced by the B602L-Fc proteome, which indicated that the B602L-Fc fusion protein could increase the half-life of antigen in mice. Those results indicate that the B602L-Fc fusion protein can target antigen to APCs by binding to the FcRI receptor of APC, to enhance the level of specific antibody production and the humoral immune response. In conclusion, B602L-Fc fusion protein can improve the humoral immunity level of the mice to a certain extent.

T lymphocytes are essential for humoral and cellular immunity. In humoral immunity, the antigen is presented to B lymphocytes, concomitantly releasing lymphokines to enhance the immune effect of B lymphocytes. In cellular immunity, specific binding with target cell interactions can destroy target cell membranes and contribute to the direct killing of target cells (48). In our research, we observed that B602L-Fc fusion protein can effectively stimulate the proliferative response of specific T-lymphocytes and the release of IL-4 and IFN-γ cytokines in cells. These results indicate that B602L-Fc protein can enhance humoral immunity and stimulate the secretion of Th1 cytokines in mice and enhance the cellular immunity level.

In brief, B602L-Fc fusion protein can enhance humoral immune response and cellular immune response to a certain extent. We proved that B602L-Fc fusion protein could up-regulate the expression of antigen presentation-related molecules and cytokines in PAMs, and that B602L-Fc fusion protein could enhance humoral and cellular immune effects in mice. These results indicate that B602L-Fc fusion protein can promote the targeted presentation and improve immune response *in vitro* and *in vivo*. Therefore, it can be speculated that the binding of the IgG Fc segment of B602L-Fc fusion protein to the FcRI receptor on APC surface, such as macrophages, can promote the activation of APCs target antigen presentation to APC and increase the activation of cytokines. Thus, enhancing the body’s humoral immunity and cellular immunity level.

Our study validated a baculovirus expression method to generate ASFV B602L and B602L-Fc fusion proteins, showing that B602L-Fc fusion proteins induce specific antibody production. However, further studies are needed to evaluate immune efficacy and challenge protection in pigs. Studies are also needed to screen more ASFV immunogenic proteins and Fc combinations and optimize the immune regimen. This study provides reasonable strategies and ideas for improving the immune effect of the ASFV subunit vaccine, which may become a promising vaccine for the development of the ASFV in the future.

## Conclusions

We successfully constructed the ASFV B602L and B602L-Fc fusion protein. The B602L-Fc fusion protein could enhance humoral immunity and cellular immunity. This study reveals that ASFV B602L-Fc fusion protein may be a promising candidate subunit vaccine against ASFV.

## Data availability statement

The original contributions presented in the study are included in the article/**Supplementary Material**. Further inquiries can be directed to the corresponding authors.

## Ethics statement

All animal experiments were approved by the Institutional Animal Care and Use Committee of Shanghai Veterinary Research Institute, China (IACUC No: SVLAC/XM-M-21104) and performed in compliance with the Guidelines on the Humane Treatment of Laboratory Animals (Ministry of Science and Technology of the People’s Republic of China, Policy No. 2006 398).

## Author contributions

YY: Formal analysis, investigation, and validation. QX: Formal analysis. Methodology and writing–review and editing. YZ: Formal analysis and investigation. ZG, and JZ: Investigation and validation. Validation. BL and YQ: Validation and writing–review and editing. KL: Validation. DS: Methodology. ZM: Conceptualization, funding acquisition, and project administration. JW: Conceptualization, formal analysis, funding acquisition, investigation, methodology, and writing– original draft. All authors contributed to the article and approved the submitted version.
