# Supplementary figures and images for: B602L-Fc fusion protein enhances the immunogenicity of the B602L protein of the African swine fever virus
Source: Front Immunol. 2023 Jun 22;14:1186299. doi: 10.3389/fimmu.2023.1186299 (PMC10324578; doi:10.3389/fimmu.2023.1186299)

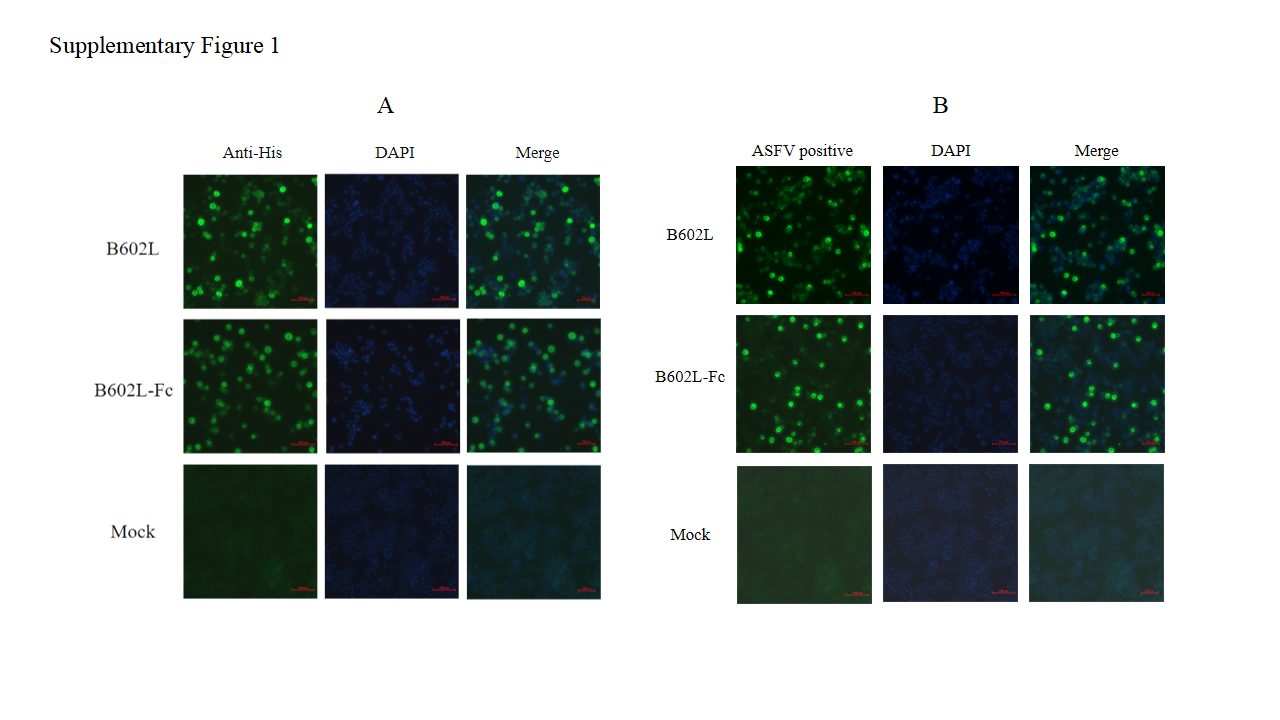

Supplement: Supplementary file 1 [file Image_1.tif]

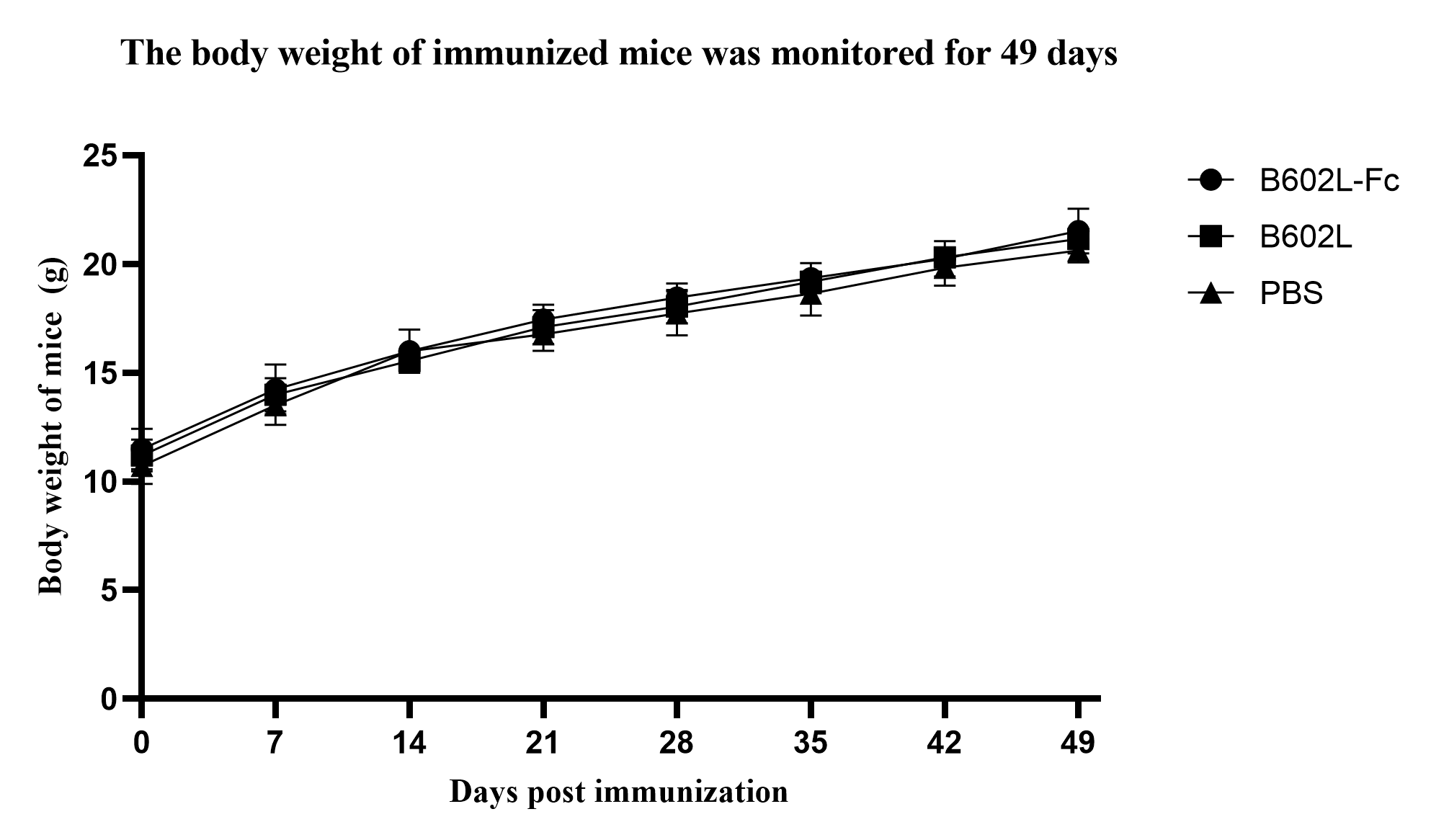

Supplement: Supplementary file 2 [file Image_2.tif]
